# The OncoAge Consortium: Linking Aging and Oncology from Bench to Bedside and Back Again

**DOI:** 10.3390/cancers11020250

**Published:** 2019-02-21

**Authors:** Paul Hofman, Nicholas Ayache, Pascal Barbry, Michel Barlaud, Audrey Bel, Philippe Blancou, Frédéric Checler, Sylvie Chevillard, Gael Cristofari, Mathilde Demory, Vincent Esnault, Claire Falandry, Eric Gilson, Olivier Guérin, Nicolas Glaichenhaus, Joel Guigay, Marius Ilié, Bernard Mari, Charles-Hugo Marquette, Véronique Paquis-Flucklinger, Frédéric Prate, Pierre Saintigny, Barbara Seitz-Polsky, Taycir Skhiri, Ellen Van Obberghen-Schilling, Emmanuel Van Obberghen, Laurent Yvan-Charvet

**Affiliations:** 1 Laboratory of Clinical and Experimental Pathology/Biobank 0033-00025, CHU Nice, FHU OncoAge, Université Côte d’Azur, 06001 Nice, France; Ilie.m@chu-nice.fr; 2 Inserm U1081, CNRS UMR7284, Institut de Recherche sur le Cancer et le Vieillissement (IRCAN), FHU OncoAge, Université Côte d’Azur, 06107 Nice, France; gael.cristofari@unice.fr (G.C.); eric.gilson@unice.fr (E.G.); veronique.paquis@unice.fr (V.P.-F.); 3 Epione Team, Inria, FHU OncoAge, Université Côte d’Azur, 06902 Sophia Antipolis, France; Nicholas.ayache@inria.fr; 4 CNRS UMR7275, Institut de Pharmacologie Cellulaire et Moléculaire, FHU OncoAge, Université Côte d’Azur, 06560 Valbonne, France; pascal.barbry@unice.fr (P.B.); philippe.blancou@unice.fr (P.B.); frederic.checler@unice.fr (F.C.); nicolas.glaichenhaus@unice.fr (N.G.); bernard.mari@unice.fr (B.M.); Seitz-polski.b@chu-nice.fr (B.S.-P.); 5 i3S Sophia Antipolis, FHU OncoAge, Université Côte d’Azur, 06560 Sophia Antipolis, France; michel.barlaud@unice.fr; 6 Centre d’Innovation et d’Usages en Santé (CIUS), FHU OncoAge, Université Côte d’Azur, 06000 Nice, France; audrey.bel@ciusante.org (A.B.); Skhiri.t@chu-nice.fr (T.S.); 7 Laboratoire de Cancérologie Expérimentale, Institut François Jacob, CEA Direction de la Recherche Fondamentale, FHU OncoAge, Université Côte d’Azur, 92265 Fontenay-aux-Roses, France; sylvie.chevillard@cea.fr; 8 Ville de Nice, Mairie de Nice, FHU OncoAge, Université Côte d’Azur, 06364 Nice, France; mathilde.demory@ville-nice.fr; 9 Nephrology Department, CHU Nice, FHU OncoAge, Université Côte d’Azur, 06001 Nice, France; esnault.v@chu-nice.fr; 10 Geriatric Unit, Centre Hospitalier Lyon Sud, Hospices Civils de Lyon, FHU OncoAge, Université Claude Bernard Lyon 1, 69310 Pierre-Benite, France; Claire.falandry@chu-lyon.fr; 11 Laboratoire CarMeN, Inserm U1060, INRA U139, INSA Lyon, Ecole de Médecine Charles Mérieux, Université Claude Bernard Lyon 1, 69921 Oullins, France; 12 Geriatric Coordination Unit for Geriatric Oncology (UCOG) PACA Est, CHU Nice, FHU OncoAge, Université Côte d’Azur, 06000 Nice, France; guerin.o@chu-nice.fr (O.G.); prate.f@chu-nice.fr (F.P.); 13 Oncology Department, Centre Antoine Lacassagne, FHU OncoAge, Université Côté d’Azur, 06189 Nice, France; joel.guigay@nice.unicancer.fr; 14 Department of Pulmonary Medicine and Oncology, CHU Nice, FHU OncoAge, Université Côte d’Azur, 06000 Nice, France; marquette.c@chu-nice.fr; 15 Département de Médecine, INSERM 1052, CNRS 5286, Centre de recherche en cancérologie de Lyon, Centre Léon Bérard, FHU OncoAge, Université Claude Bernard Lyon 1, 69008 Lyon, France; pierre.saintigny@lyon.unicancer.fr; 16 Laboratory of Immunology, CHU Nice, FHU OncoAge, Université Côte d’Azur, 06200 Nice, France; 17 CNRS, Inserm, iBV, Centre Antoine Lacassagne, FHU OncoAge, Université Côte d’Azur, 06108 Nice, France; ellen.van-obberghen@unice.fr; 18 CNRS, LP2M, FHU OncoAge, Université Côte d’Azur, 06107 Nice, France; emmanuel.Van-obberghen@unice.fr; 19 Inserm U1065, Centre Méditerranéen de Médecine Moléculaire (C3M), FHU OncoAge, Université Côte d’Azur, 06200 Nice, France; laurent.yvan-charvet@unice.fr

**Keywords:** aging, cancer, optimization, research, education, elderly, well-being

## Abstract

It is generally accepted that carcinogenesis and aging are two biological processes, which are known to be associated. Notably, the frequency of certain cancers (including lung cancer), increases significantly with the age of patients and there is now a wealth of data showing that multiple mechanisms leading to malignant transformation and to aging are interconnected, defining the so-called common biology of aging and cancer. OncoAge, a consortium launched in 2015, brings together the multidisciplinary expertise of leading public hospital services and academic laboratories to foster the transfer of scientific knowledge rapidly acquired in the fields of cancer biology and aging into innovative medical practice and silver economy development. This is achieved through the development of shared technical platforms (for research on genome stability, (epi)genetics, biobanking, immunology, metabolism, and artificial intelligence), clinical research projects, clinical trials, and education. OncoAge focuses mainly on two pilot pathologies, which benefit from the expertise of several members, namely lung and head and neck cancers. This review outlines the broad strategic directions and key advances of OncoAge and summarizes some of the issues faced by this consortium, as well as the short- and long-term perspectives.

## 1. Introduction

**Figure 1:**
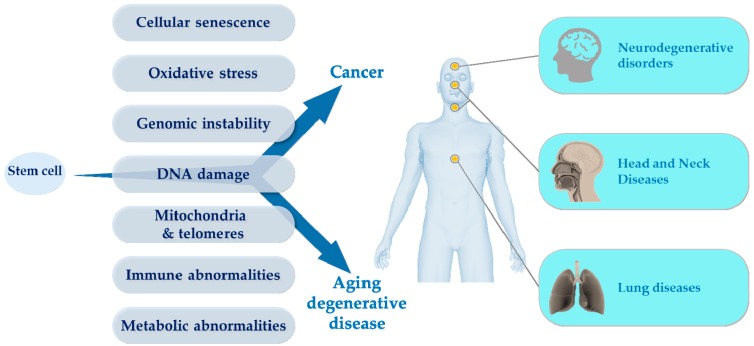
Common mechanisms that drive degenerative diseases and cancer. OncoAge is dedicated to three major pathologies: thoracic diseases (tumoral or non-tumoral), head and neck and thyroid pathologies, and neuromuscular degenerative disorders.

Chronological age is the most important single risk factor for the development of a variety of cancers and chronic diseases that account for the majority of societal morbidity, mortality, and public health costs. Recent findings suggest that changes in certain basic biological processes are shared in physiological aging, cancer, and degenerative pathologies [1,2]. Importantly, similar processes can be altered in diseases as diverse as cancer, neurodegeneration, cardiovascular disorders, chronic obstructive pulmonary disease (COPD), osteoarthritis, and diabetes, to name a few. For instance, at the cellular level, the accumulation in tissues of senescent cells (permanent cell cycle arrest in response to various types of stress or tissue remodeling) emerges as an important contributor to aging and age-related pathologies, through both cell autonomous and non-autonomous mechanisms driving inflammation, immunosenescence, and tissue degeneration [3,4]. Therefore, a key challenge now is to rapidly improve our knowledge on the biological processes in common that lead to malignant transformation and degenerative pathologies [1,5,6,7]. From a cellular standpoint, the mechanisms that drive degenerative diseases and cancer are shared at an initial phase (e.g., during the accumulation of senescent cells), before adopting a particular direction and specific genetic and epigenetic modifications that orient cells toward distinct fates (e.g., escape of cellular checkpoints for cancer cells) [1,5,6,7,8]. Thus, schematically, degenerative aging and cancer can be considered as two sides of the same coin, involving many common fundamental biological mechanisms (Figure 1).

Hence, the progressive degeneration of tissues can lead to transformation into cancer after activation of chronic inflammation and immunosenescence [9,10,11,12]. Finally, from an epidemiological standpoint, the risk of emergence and incidence of most cancers increase with the age of the population [13,14,15].

Although cancer and aging biology are closely related, they are often investigated separately. Thus, whereas a number of fundamental and translational research centers or institutes worldwide have oriented their research in the direction of aging, only a few of them have really focused their studies on the links between aging and cancer. This is the case for the Institute for Research on Cancer and Aging, Nice (IRCAN) in France, which bases its overarching strategy on combining the research developed by scientists and physicians on cancer and aging mechanisms (https://www.ircan.org). It is within this context that the OncoAge consortium was launched in Nice to facilitate the transfer of this growing knowledge on cancer and aging to medical innovation and current medical practice. This consortium was certified and recognized in 2015 as a Hospital-University Federation (HUF) by AVIESAN (https://www.aviesan.fr; https://www.oncoage.org). The global aim of the HUF program in France is to develop excellence within the university hospitals by targeting medical topics optimizing care, research, and education in these subject areas (https://www.aviesan.fr). In short, OncoAge is a HUF based on the expertise of medical and scientific teams oriented toward cancer pathologies associated with aging. The key aim of OncoAge is to improve the care of elderly patients, in particular those with cancer, to set up research projects, and develop training and educational programs in this domain (https://www.oncoage.org). These efforts should not only deepen our understanding of the mechanisms underlying cancer and aging, but also improve the daily well-being of the patients.

The aging of the world’s populations has progressively modified the profile of the most frequent diseases [13]. While infectious and cardiovascular disorders have until recently been the most frequent, and resulted in the highest number of deaths around the globe, considerable progression towards an increase in the number of certain cancers and diseases linked to aging has been observed in recent years. According to epidemiological predictions, these diseases will be among the most common in 2030, in both industrialized and non-industrialized countries. Among them, lung cancer will be the fifth cause of death in 2030, whereas according to the Global Burden of Disease (GBD), COPD is already now the third leading cause of death worldwide, a progression WHO had not predicted to occur until 2030 [16,17].

In this context, it is crucial to rapidly advance the molecular understanding of genetic and epigenetic mechanisms, as well as immune and metabolic abnormalities leading to the development of cancers associated with age, and to improve the care and well-being of patients with cancers that have become chronic and often invalidating. This has generated an urgent need to address many new challenges in translational projects in this field [18].

Importantly, elderly patients with lung cancer and head and neck cancer (HNC) are rarely enrolled in clinical trials, particularly in phase 1, and even less so in dedicated trials in curative or palliative settings. As an example, no standards of treatment exist for these populations, and frail elderly lung and HNC patients may be over-treated with a risk of increased toxicity while fit patients may be proposed for suboptimal treatment. It is, therefore, crucial to develop and evaluate appropriate treatments by enrolling elderly patients with cancer in a higher number of therapeutic trials. Beyond research-related concerns, OncoAge faces epidemiologic and environmental issues such as the procurement of well-controlled demographic data and the means of measuring air pollutants according to geolocalization of the patients in the Alpes-Maritimes area. Moreover, questions concerning costs (obtaining funding from public and private sources) and organization (steering multicenter efforts in the same direction) must be anticipated and managed to assure the sustainability of the consortium in the near years.

The genesis and objectives of the OncoAge consortium since its creation in 2015 at the Côte d’Azur University (Nice, France), its first accomplishments, and its future perspectives are described below.

## 2. OncoAge: The Origin of the Project

OncoAge was established in France after acceptance and certification by AVIESAN, subsequent to a national tender for HUF proposals (https://www.aviesan.fr). The application called for unique and original projects covering an aspect of health for which a program optimizing the healthcare of patients, research, and teaching in the specified domain could be addressed. The HUF OncoAge project was submitted in 2015 and selected by AVIESAN after the representatives of the project were examined by an international committee.

## 3. OncoAge at the Côte d’Azur University: Why?

**Figure 2:**
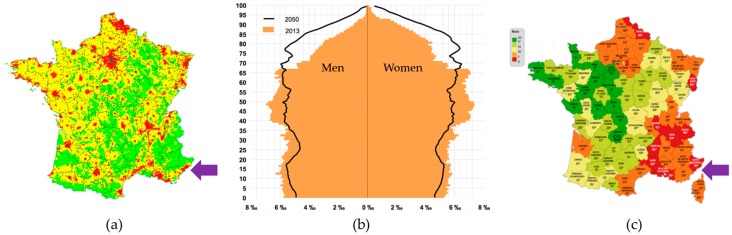
(**a**) High population density within the Alpes-Maritimes area (purple arrow) (INSEE data). (**b**) Age pyramid within the Alpes-Maritimes area in 2013 and pre-visions for 2050 (INSEE data). (**c**) Air pollution levels (in red, very high air pollution) within the Alpes-Maritimes area (purple arrow; www.prevair.org).

The choice of setting up a HUF within the Nice Hospital of the University Côte d’Azur was motivated by several aspects, in particular, based on epidemiological arguments (Figure 2).

According to the data from the National Institute of Statistics and Economic studies (“INSEE”), in contrast to other regions in France, the population of the Alpes-Maritimes region, where the HUF is located, is populated by a high number of elderly people (>65 years old) (https://www.insee.fr). This region has a population density above the average in France, and which is increasing yearly (https://www.insee.fr). Indeed, the Nice University Hospital already treats many patients above 65 years of age, and this number is expected to increase. In this context, due to the global aging of the world population, it is estimated that the number of elderly patients admitted to this healthcare center today is similar to that which will be observed in most hospitals in the world in 2030. The high incidence of lung cancer and the above average level of atmospheric pollution in the Alpes-Maritimes region, as compared to national levels, were among the other reasons that motivated the decision to develop the OncoAge project (Figure 2). Moreover, no other HUF in France has focused on aging and cancer. Therefore, we felt that this important issue should be developed within this geographical area. Finally, the presence of IRCAN, a research center focusing on the mechanisms linking cancer and aging, provides a unique opportunity to associate the most recent biological discoveries with the health-oriented aims of OncoAge.

## 4. OncoAge at the Côte d’Azur University: How?

OncoAge efforts are based, for the greater part, on a limited number of “pilot” pathologies that were selected within the Nice Hospital, taking into consideration the following parameters: (i) optimal organization of the hospital sectors in the concerned domains and the potential recruitment of patients, (ii) activities of the university (publications and teaching) of the hospital departments, (iii) the organized clinical and/or biological databases, (iv) clinical and translational research performed in collaboration with scientists of the teams studying fundamental research in the specified subjects. For this, three major pathologies were initially chosen to set up the foundations of OncoAge: thoracic diseases (tumoral or non-tumoral), head and neck and thyroid pathologies, and neuromuscular degenerative disorders. Transversal studies were initiated to reinforce the fundamental knowledge on these medical questions, combining various (epi)genomics, immunology, metabolism, and artificial intelligence (AI) approaches since these topics are particularly important for aging and cancer [1,19,20,21,22,23,24,25,26,27]. Actions that support common structures have been established, such as innovative programs and connection to the silver economy (https://www.france-silvereco.fr/notre-observatoire/tableau-de-bord-de-la-filiere), technological platforms (including geriatric screening tools to identify elderly cancer patients who could benefit from comprehensive geriatric assessment), and new biorepository space, training and education, dissemination of knowledge and information [28,29,30,31,32,33]. The participants working on these different aspects (work package leaders) interact in concert with the unique aim of building a dynamic, collaborative network.

**Figure 3:**
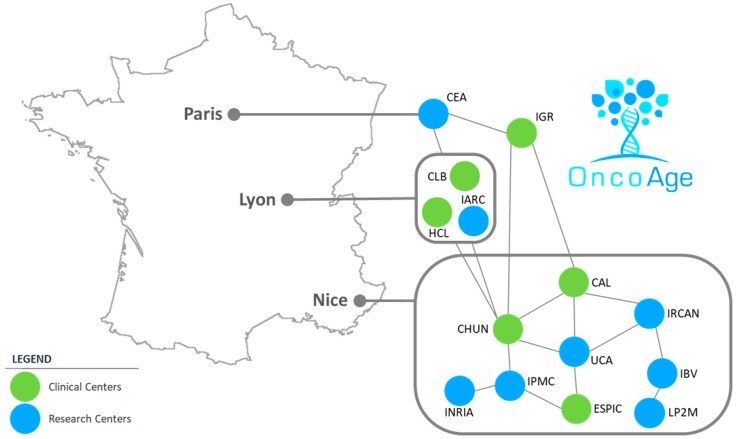
The OncoAge consortium members. CEA: Centre d’Energie Atomique; IGR: Gustave Roussy Institute; CLB: Léon Bérard Comprehensive Cancer Center; IARC: International Agency for Resarch on Cancer; HCL: Hospices Civils de Lyon; CAL: Antoine Lacassagne Comprehensive Cancer Center; CHUN: Centre Hospitalier Universitaire de Nice; UCA: Université Côte d’Azur; IRCAN: Institut of Research and Aging, Nice; IBV: Institut de Biologie Valrose; LP2M: Laboratoire de PhysioMédecine Moléculaire; ESPIC: Etablissement de Santé Privé d’Intérêt Collectif Hôpitaux Pédiatriques de Nice Centre Hospitalier Universitaire Fondation Lenval; IPMC: Institut de Pharmacologie Cellulaire et Moléculaire; INRIA: Institut National de Recherche en Informatique et en Automatique.

In addition to several departments of the Nice University Hospital, the OncoAge consortium brings together a number of institutes of the Côte d’Azur University (UCA), such as the Antoine Lacassagne Comprehensive Cancer Center (CAL), the Etablissement de Santé Privé d’Intérêt Collectif (ESPIC) Hôpitaux Pédiatriques de Nice Centre Hospitalier Universitaire Fondation Lenval, and many teams of different research centers (Institut of Research and Aging, Nice (IRCAN), Centre Méditerranéen de Médecine Moléculaire (C3M), Institut de Biologie Valrose (iBV), Institut de Pharmacologie Cellulaire et Moléculaire (IPMC), Laboratoire de PhysioMédecine Moléculaire (LP2M), and Institut National de Recherche en Informatique et en Automatique (Inria)). OncoAge is not only composed of different stakeholders belonging to the Côte d’Azur University but also of teams of the Lyon University (Lyon University Hospital; Hospices Civils de Lyon; HCL; and Leon Bérard Comprehensive Cancer Center (CLB) and of the International Agency for Research on Cancer (IARC, Lyon, France), the Centre d’Energie Atomique (CEA; Fontenay aux Roses, France), and the Gustave Roussy Institute (IGR; Villejuif, France) (Figure 3).

The governance of OncoAge is provided by a strategic committee and a scientific council (https://www.oncoage.org). To ensure proper functioning and to benefit from expert advice, an international scientific advisory board (SAB) composed of medical and scientific opinion leaders in the field of cancer and aging has been set up (https://www.oncoage.org).

## 5. OncoAge: Main Objectives

The overarching objective of OncoAge is to create and foster a network of expertise, and to develop collaborations and projects to improve the healthcare of elderly patients with cancer. To attain these objectives, a number of initiatives have been undertaken: (i) definition of different indicators of tracking (using publications), (ii) development of clinical and translational research projects funded by regional, national, and international bodies, (iii) introduction into the university lectures of themes developed by OncoAge, (iv) organization of workshops and conferences, and (v) communication of information to the general public and the creation of a dedicated website. These different initiatives put forward by OncoAge were first evaluated by the SAB of OncoAge in 2015, and found to be highly appropriate.

## 6. OncoAge: Main Results After Three Years of Existence (2015–2018)

Several key accomplishments both at the level of clinical and translational research projects and at the level of the structuring and dissemination of information were achieved. The clinical expertise in HNC within OncoAge is highlighted by the ELAN (ELderly Head and Neck cancer) program. These clinical studies on curative or palliative personalized treatment of elderly head and neck cancer patients after geriatric assessment (Elan Geriatric Evaluation, EGE) currently represent the only multi-center therapeutic trials dedicated to this group of patients worldwide [34,35,36]. Studies were completed in 2018 and results will be presented in 2019. Moreover, an early phase clinical trial unit was set up in the Comprehensive Cancer Center Antoine Lacassagne to favor the emergence of therapeutic innovations.

Since 2015, OncoAge has been associated with the publication of 86 scientific articles referenced in NCBI PubMed (the “FHU OncoAge” was listed with the author’s affiliation). For example, some publications are related to epidemiological, clinical, and translational research projects made in lung cancer and COPD, such as lung cancer screening and assessment of biomarkers [37,38,39,40,41,42,43,44,45,46]. Recently, a new project was accomplished by physicians from the Nice University Hospital and researchers belonging to the “Institut National de Recherche en Informatique et en Automatique” (Inria) who aimed to develop a lung cancer screening program based on the integration of three signatures: clinical data (leading to better risk factor assessment), chest low dose CT scan (by using computer-aided diagnosis), and biological blood signatures [47]. Since the HUF OncoAge was established, a strong partnership between oncologists and geriatricians belonging to the consortium was set up in order to optimize the care of the elderly cancer population. In this context, a large comprehensive geriatric assessment program using a multidimensional interdisciplinary diagnostic process was rapidly developed [48].

Other specific studies concern the head and neck pilot pathology [49,50]. Several scientific projects managed by leaders of OncoAge were financed by different organizations, including the Institut National du Cancer (the French NCI), l’Agence Nationale de la Recherche, la Fondation de l’Association de la Recherche contre le Cancer, le Cancéropôle Provence Alpes Côte d’Azur” (PACA), and the Infrastructure en Biologie Santé et Agronomie” (IBiSA). A master’s program on “Biobanks and Complex Data Management” was set up through the association of the Côte d’Azur University and the Nice Hospital (https://MSc biobanks-complex-data) [51]. The Laboratory of Clinical and Experimental Pathology within OncoAge has been selected by the European Society of Pathology to serve as an advanced training center for molecular pathology with an emphasis on liquid biopsy. Since 2015, the master’s program has enrolled students from all around the world and so far, three classes of students have been trained.

This master’s is supported by the biobank of the Nice University Hospital (BB-0033-0025), which has benefited since 2015 from new infrastructures and developments (http://univ-cotedazur.fr/en/education/informations-utiles/les-informations-utiles/biobanks-complex-data/#.XGlsb7jjJ4E) [51].

This biobank has integrated the technological platform of OncoAge and the biological specimens associated with the clinical data and is available to the teams of OncoAge after a material transfer agreement has been signed. The visibility of OncoAge has been ensured through the creation of a website and the organization of several symposiums, including the first joint meeting on lung cancer associating the MD Anderson Cancer Center and the HUF OncoAge (https://www.oncoage.org/news-and-events-2/3/). Moreover, recently, on behalf of the HUF OncoAge, different actors of the consortium have had the opportunity to participate in the writing of a next Encyclopledia of Aging and Population Aging, edited by Springer, which will be available at the end of 2019. The HUF OncoAge will lead the “Cancer and Treatment” section of this encyclopedia.

The different actions accomplished by OncoAge were favorably evaluated by the SAB of OncoAge at the end of 2018.

## 7. OncoAge: Current Developments and Perspectives

A number of perspectives have been envisioned for the short term (2020). First, broadening the pathologies that OncoAge intends to investigate, in particular, skin cancers (including melanoma), will be integrated in 2019. New clinical–biological collections will be built, either as a complement to existing collections or from a new population of patients. Concerning the complementary collections (from patients with lung cancer and chronic obstructive pulmonary disease), the samples will include urine, total blood, and peripheral blood mononuclear cells (PBMCs). A new collection of blood (plasma, PBMCs, and total blood) obtained from healthy individuals older than 80 years and residents of the Alpes-Maritimes region will be assembled. Finally, a collection of bronchial and transthoracic biopsies (tissues fixed and paraffin embedded) obtained from lung cancer patients will be set up for future translational research projects.

Several other objectives have been defined. Amongst these, an international master’s degree on aging, dedicated to researching questions concerning aging and associated diseases, should be created. Further, a national project implicating private–public partners targeting innovation in the domain of aging should initiate several national and international collaborations (http://www.agence-nationale-recherche.fr/). Finally, new clinical trials, all with translational studies (including mechanisms of resistance to immunotherapy), have been launched or are in advanced phases of discussion with academic institutional groups and pharmacological companies. Indeed, immunotherapy in elderly patients has become a promising treatment alternative and put in the limelight [52,53,54,55,56]. In this context, the development and assessment of biomarkers of senescence will be associated with clinical trials thanks to the biobank (BB-0033-00025) and the OncoAge research teams [57,58]. These studies will benefit from the acquisition by OncoAge of technical platforms of state-of-the-art equipment and AI-based software (HALO AI^TM^, Indica Labs, London, UK) for high-speed whole slide imaging and quantitative multiplexing.

## 8. Conclusions

The most prominent feature of aging is a gradual deterioration/loss of cells that is associated with organ dysfunction and the rise of age-related chronic pathologies. Amongst these, cancer stands out as its occurrence significantly increases with age and has a devastating human and public health cost. Deciphering the clinical features, the biological markers, and the lifestyle and environmental factors that are shared between common chronic age-related pathologies and cancer should lead to the development of new clinical approaches, including the validation of surrogate biomarkers of frailty and predisposition. A deeper understanding of the common mechanisms involved both in aging and cancer is expected to considerably improve our knowledge on how to prevent age-related pathologies and how to optimize the care of elderly patients. In this context, OncoAge is a unique consortium composed of more than 1000 participants and actors located in Nice, Lyon, and Paris with the exclusive ambition of working together on clinical–biological and medical–scientific projects that aim to improve the care of elderly patients with cancer. This consortium has actively developed translational and clinical projects and has created innovation in the domain of geriatric oncology. The increase in the age of the world’s populations has created new urgent demands on healthcare, as well as major strategic and economic issues. Improving the autonomy of elderly patients with cancer, avoiding repeated and long hospitalizations, performing early screening for certain cancers, and predicting, as well as preventing complications, are all objectives set out by OncoAge. Moreover, understanding the relationships between the aging phenomenon and cancer is a timely and multifaceted challenge where high-level research efforts in medicine, genomics, and biology have to be combined with societal approaches focused on individuals. In this context, OncoAge has designed and made operational an original holistic approach combining genotype and phenotype analyses of the aging and cancer processes.

To conclude, our expert multipronged approach is consolidated by the enthusiasm of the many physicians and scientists of several leading hospitals and strong research centers and warrants the future of OncoAge.
